# Effects of training flights of combat jet pilots on parameters of airway function, diffusing capacity and systemic oxidative stress, and their association with flight parameters

**DOI:** 10.1186/s40001-024-01668-z

**Published:** 2024-02-05

**Authors:** Janina Bojahr, Rudolf A. Jörres, Angelika Kronseder, Frank Weber, Carla Ledderhos, Immanuel Roiu, Stefan Karrasch, Dennis Nowak, Daniel Teupser, Christian Königer

**Affiliations:** 1 Institute and Outpatient Clinic for Occupational, Social and Environmental Medicine, Comprehensive Pneumology Center Munich (CPC-M), Member of the German Center for Lung Research (DZL), University Hospital, LMU Munich, Munich, Germany; 2 Federal Armed Forces Hospital, Lesserstr. 180, 22049 Hamburg, Germany; 3 Air Force Centre of Aerospace Medicine, Fuerstenfeldbruck, Cologne, Germany; 4 74th Tactical Air Wing of the German Air Force, Neuburg, Germany; 5 Institute for Laboratory Medicine, University Hospital, LMU Munich, Munich, Germany; 6 Occupational Medicine Department, Medical Support Center Munich, Munich, Germany

**Keywords:** Lung function, Diffusing capacity, Exhaled nitric oxide, Oxidized guanosine species, Jet fighter pilots, G-forces, Life support system, Positive pressure breathing

## Abstract

**Background:**

Fighter aircraft pilots are regularly exposed to physiological challenges from high acceleration (G_z_) forces, as well as increased breathing pressure and oxygen supply in the support systems. We studied whether effects on the lung and systemic oxidative stress were detectable after real training flights comprising of a wide variety of exposure conditions, and their combinations.

**Methods:**

Thirty-five pilots of the German Air Force performed 145 flights with the Eurofighter Typhoon. Prior to and after flight lung diffusing capacity for carbon monoxide (DL_CO_) and nitric oxide (DL_NO_), alveolar volume (V_A_), and diffusing capacities per volume (K_CO_, K_NO_) were assessed. In addition, the fractional concentration of exhaled nitric oxide (FeNO) was determined, and urine samples for the analysis of molecular species related to 8-hydroxy-2’-deoxyguanosine (8-OHdG) were taken. For statistical analysis, mixed ANOVA models were used.

**Results:**

DL_NO_, DL_CO_, K_NO_, K_CO_ and V_A_ were reduced (*p* < 0.001) after flights, mean ± SD changes being 2.9 ± 5.0, 3.2 ± 5.2, 1.5 ± 3.7, 1.9 ± 3.7 and 1.4 ± 3.1%, respectively, while FeNO decreased by 11.1% and the ratio of 8-OHdG to creatinine increased by 15.7 ± 37.8%. The reductions of DL_NO_ (DL_CO_) were smaller (*p* < 0.001) than those of K_NO_ (K_CO_). In repeated flights on different days, baseline values were restored. Amongst various flight parameters comprising G_z_-forces and/or being indicative of positive pressure breathing and oxygenation support, the combination of long flight duration and high altitude appeared to be linked to greater changes in DL_NO_ and DL_CO_.

**Conclusions:**

The pattern of reductions in diffusing capacities suggests effects arising from atelectasis and increased diffusion barrier, without changes in capillary blood volume. The decrease in exhaled endogenous NO suggests bronchial mucosal irritation and/or local oxidative stress, and the increase in urinary oxidized guanosine species suggests systemic oxidative stress. Although changes were small and not clinically relevant, their presence demonstrated physiological effects of real training flights in a modern 4th generation fighter jet.

**Supplementary Information:**

The online version contains supplementary material available at 10.1186/s40001-024-01668-z.

## Introduction

Pilots of jet-powered combat aircrafts experience physical stress, for example in the form of acceleration forces (G-forces) or the positive pressure breathing (PPB) of the life support system. The positive G_z_ acceleration directed towards the head (in units of gravitational acceleration *g*) is of particular interest, being that with the most significant impact on the pilots’ body during flight, as it leads to blood pooling in the legs and away from the brain . It occurs for instance, when pilots fly tight curves during tactical manoeuvring. The “natural” G_z_-tolerance for trained personnel without using any support or protection system is in the range of 5 G_z_, depending on the g-onset rate. It is for example 5.4 ± 0.9 G_z_ with a gradual onset rate of 1 G_z_/s and 4.5 ± 0.6 G_z_ with an onset rate of 2 G_z_/s [1]. Beyond this, the acceleration inevitably leads to loss of consciousness (G-LOC). Due to their much higher agility and associated G_z_-onset rates, the prodromal stage of "grayout" known from earlier aircraft types is no longer present as a warning sign for the pilots [2]. For example, in the Eurofighter Typhoon, a typical fourth-generation fighter jet, the G_z_-onset rate can reach values up to 15 G_z_/s [3]. Therefore, elaborate measures including anti-G pressure suits and pressure breathing for G-protection (PBG) protect pilots from G-LOC. Both are part of the Eurofighter’s environmental control system (ECS). Whilst the pressure suit acts against blood pooling into the legs, breathing under positive pressure combined with oxygenation of inhaled air serves to prevent adverse effects such as cerebral hypoxemia, thereby increasing G-tolerance.

These interventions raise the question as to whether the support systems themselves may lead to  adverse physiological effects. In intensive care medicine, it is known that increased airway pressures can have detrimental effects on the lung [4, 5] causing barotrauma and ventilation-induced lung injury in extreme cases [6, 7]. Furthermore, the dry inhaled air may induce irritation of the upper respiratory tract [8], and the increased partial pressure of oxygen and its variation over time can lead to resorption atelectasis [9] in the lung periphery, or the formation of oxygen radicals leading to both local, and systemic, oxidative stress and DNA damage [10, 11].

There are several approaches to detect such hypothetical effects via non-invasive—and thus feasible and easily acceptable—procedures. Alterations in the alveolar compartment can be assessed via the combined diffusing capacity (DL_NO_, DL_CO_) for nitric oxide (NO) and carbon monoxide (CO) [12], and the alveolar volume (V_A_) can be determined via helium dilution. Effects on the upper respiratory tract might be detected via alterations in the fractional exhaled nitric oxide (FeNO) level [13], and systemic oxidative stress via the concentration of 8-hydroxy-2’-deoxyguanosine (8-OHdG) in urine [14, 15]. It has to be considered that invasive procedures such as blood sampling could not be performed due to safety restrictions.

Some of these outcomes have already been addressed in studies using centrifuge experiments [16]. There is, however, a lack of data (a) referring to real-life conditions with a broad spectrum of realistic flight parameters, (b) including a large number of flights and pilots, (c) involving repeated flights on the same day or on different days, and (d) comprising the panel of outcome variables described above. Besides providing unique data on the pattern of effects occurring under real flight conditions, such data might also provide suggestions regarding  possible health risks. Based on these considerations, we performed measurements before and after real training flights in German combat fighter pilots under a wide variety of flight conditions.

## Material and methods

### Study design

The study was performed in cooperation with a tactical fighter wing of the German Air Force. Due to its organization, the squadron allowed for measurements twice a day in the same pilots. As the assessments had to be integrated into the routine schedule to ensure that the study was performed under real-life conditions, the available time window of assessments prior to and after the flights was restricted to 15 min. To satisfy these requirements, the final protocol was established in close cooperation with the local commander and the flight surgeon. It was approved by the Ethical Committee of the Bavarian Medical Association, as well as the local personal council, and all participants gave their informed consent. Data collection took place between March and June 2021.

To achieve maximum statistical power, the design included measurements before and after each flight. In single cases, post-measurements had to be cancelled due to organizational reasons; these flights were excluded from the analysis. Depending on the location of the airplanes and their specific tasks, measurements were performed in two squadron buildings next to the pilots' changing rooms, with the aim to minimize the time interval between flight and measurement. Pre-flight measurements were performed after donning the pressure suit and about 30 min before take-off. After the completion of pre-flight measurements, pilots were transferred to the shelter, where their airplane was being prepared for take-off. Regarding post-flight assessments, pilots were asked to attend the site of measurements as soon as possible, before taking off their pressure suit.

Each assessment comprised the following measurements. First, the level of exhaled nitric oxide (FeNO) was determined to prevent interference from the other assessments; measurements were performed at least twice. This was followed by at least two measurements of the combined diffusing capacity for inhaled NO and CO (DL_NO_ and DL_CO_). In the time between these repeated measurements, a symptom questionnaire was answered. Afterwards, a urine sample was supplied by the pilot. In preliminary assessments, an average duration of 11 min per panel of measurements was found, which thus could be well-performed in the planned 15-min time slots. For data analysis, all relevant time points including those of measurements were recorded.

### Recruitment of participants

The objective of the study was explained to the pilots in a collective briefing, and information and consent sheets were distributed. Further details of the assessments were given in a subsequent presentation. In close cooperation with the Operations officer, the use of the available flight-slots was established, according to which theoretically up to two pilots could be measured in each of the two available flight rounds per day.

### Assessments

#### Measurement of fractional exhaled nitric oxide (FeNO)

We used a chemiluminescence analyzer (CLD 88 sp, Ecomedics AG, Duernten, Switzerland) with appropriate breathing equipment. Participants were asked to exhale into the mouthpiece at a constant flow rate of 50 mL/s as recommended [17]. For a valid measurement, at least two manoeuvres had to be performed. If plateau values agreed within 10% of each other, the measurement was considered as valid and the mean of both values was taken, otherwise it was repeated [17]. In comparison with the common use of FeNO, in the present study we did not expect Th-2-related increases in FeNO but a possible decrease due to drying/dehydration of the airway mucosa from inhaled dry air and/or oxidative stress from inhaled oxygen, with scavenging of endogenous NO.

#### Diffusing capacity for inhaled NO and CO

For the determination of diffusing capacity, commercial equipment (Masterscreen PFT, Vyaire Medical, Höchberg, Germany) with a test gas mixture containing 40–50 ppm NO, 0.3% CO and 9.3% helium was used, and manoeuvres were performed according to ATS/ERS guidelines [18–20]. To ensure quality, at least two measurements were performed, with a time interval of at least 4 min in between. We determined the diffusing capacity (transfer factor) for nitric oxide (DL_NO_) or carbon monoxide (DL_CO_), their ratio (DL_NO_/DL_CO_), the alveolar volume (V_A_), as well as diffusing capacity per alveolar volume (transfer coefficient) for nitric oxide (K_NO_) and carbon monoxide (K_CO_). In these assessments, we expected potential reductions in alveolar volume (V_A_) from flight-induced atelectasis, moreover a decrease in DL_NO_ due to a reduction in V_A_ and/or changes of alveolar surface properties, for example from fluid imbalance due to G-forces, moreover additional changes in DL_CO_ indicative of alterations in lung perfusion.

#### Symptom questionnaire

The questionnaire comprised 100 mm visual analogue scales (VAS), reaching from "not at all" to "extremely strong", for each of 10 items. These included the 9 symptoms: difficulty of breathing, earache, headache, eye discomfort, cough, sputum, scratchy throat, dry mouth, dry nose, with the option for a 10th item in case of an additional symptom.

#### Urine samples and determination of oxidized guanosine species

To determine oxidized guanosine species, urine samples were collected shortly after completion of the lung function tests. If possible, an additional sample was collected within 2–4 h after landing. Samples were taken from midstream urine and immediately stored at − 20 °C for later analysis. They were analyzed for their creatinine content on a Roche Cobas 8000 analyzer, as well as for oxidized guanine species including 8-hydroxy-2’-deoxyguanosine (8-OHdG), 8-hydroxyguanosine and 8-hydroxyguanine, using a high-sensitive DNA/RNA Oxidative Damage ELISA Kit (#589320, Cayman Chemical, Michigan, USA). For simplicity, these species are collectively termed as 8-OHdG. For each sample, triplicate measurements in each of two different dilutions were performed. A coefficient of variation within each triple of < 10% and a difference of concentrations deriving from the two dilutions of < 20%, were required for validity. For data analysis, the concentration of 8-OHdG was normalized to that of creatinine in each respective sample. In the present study, 8-OHdG was included as a potential systemic marker of oxidative stress. An overview of the protocol is given in Additional file 1: Fig. S1.

### Analysis of exposure and outcome parameters

The analysis had three aims: (1) describing the range of flight exposure parameters for real-flight conditions, (2) assessing changes in outcome parameters via comparison of pre- and post-flight values, (3) identification of associations between outcome and exposure parameters. Exposure parameters comprised the duration of flight, moreover G_z_-force and flight altitude that triggered the action of the support systems (G-suit, mask). This determined time and degree of pressure breathing and oxygenation of inhaled air, apart from the fact that overall the inhaled air was of low humidity compared to ambient air at the ground. Pressure and oxygenation were adjusted to the flight parameters via specific algorithms, whereby pressure was a function of  altitude and G-forces, starting at + 4G_z_, with a maximum of 80 mbar during 9-G_z_-manoeuvres [21]. The applied oxygen level was a function of cabin altitude , which depends on flight altitude in a nonlinear manner [22].

Pressure and oxygenation through the mask were not directly recorded; however, as surrogate markers we used altitude and G-forces that were its determinants and recorded over time. These recordings comprised their minima and maxima in each minute of the flight, from which exposure indices such as their quantiles, mean values and standard deviations, as well as numbers of occurrences in excess of cut-off values could be calculated.

### Statistical analysis

For data description, mean values and standard deviations (SD), or standard errors of mean (SEM), or median values and quartiles, or numbers and percentages were chosen, depending on the data type. For FeNO and the ratio DL_NO_/DL_CO_, geometric mean values and standard deviations were computed, the latter representing multiplicative variability factors. Correspondingly, these two variables were included as logarithmically transformed values in the statistical analyses. Statistical analyses were performed with linear models, comprising the pilot as random factor, and as fixed factors and covariates either a pre-post indicator, or the continuous exposure measures, or their binary reductions (see results). Post-hoc comparisons were performed according to Duncan, correlation analyses via Pearson’s linear or Spearman’s rank correlation analysis, depending on the type of variable.

In the absence of data obtained under the multiple combinations of real-life flight conditions, no precise information on the range of expected changes and the variability was available. In other clinical exposure studies, we had observed significant effects regarding FeNO or diffusing capacity with sample sizes of *n* = 20 to 30 [23, 24], and centrifuge data showed significant effects on lung function already in small numbers of simulated flights [16]. Based on this, we aimed for a minimum number of 20 pilots with a minimum of 4 flights per pilot. Statistical significance was assumed for *p* < 0.05; *p* values below 0.1 are presented as tendency. All analyses were performed using SPSS (Version 26, IBM Corp., Armonk, NY, USA).

## Results

### Study population characteristics

Overall, 145 flights performed by 35 pilots with pre- and post-assessments were available (Table 1); 64 of them took place before noon (0900 hrs–1200 hrs.), 68 in the afternoon (1400 hrs– 1800 hrs), and 13 in the evening (1800 hrs–2200 hrs). All pilots were male and between 25 and 57 years of age. None were active smokers, 6 had a history of seasonal allergic rhinitis. The distribution of frequencies of flights is given in Table 2; 22 pilots performed at least 4 flights, the maximum number being 10. On average, post-flight measurements occurred a median time (quartiles, range) of 28 (23, 32.5; 13 to 54) min after landing and 184 (154, 231.5; 88 to 536) min after pre-flight measurements, depending on the duration of the flight and the schedule into which the assessment had to be implemented.

**Table 1:** Characteristics of the 35 pilots who participated in the study

Variable	Mean ± SD or number	Minimum–Maximum
Age (years)	36.3 ± 8.2	25 – 57
Height (cm)	180.6 ± 5.9	172 – 195
Weight (kg)	81.1 ± 9.0	67 – 102
BMI (kg/m^2^)	24.8 ± 2.1	21.9 – 30.6
Smoking status (never/ex)	(32/3)	-
History of allergy (yes)	6	-
VC (L)	5.44 ± 0.61	4.29 – 6.96
VC (% predicted, GLI)	98.7 ± 9.2	83.0 – 133.8
FeNO (ppb)*	17.8 (1.81)	6.6 – 56.5

Numbers or mean values and standard deviations (SD) are given

*VC*  vital capacity determined in the single-breath manoeuvre within the assessment of diffusing capacity, *FeNO*  fractional concentration of exhaled nitric oxide. Predicted values were taken from those for forced vital capacity of the Global Lung Function Initiative (GLI). *BMI*  body mass index. *Geometric mean and standard deviation (in parentheses, to be interpreted as variability factor of the geometric mean)

**Table 2:** Number of flights per pilot and number of pilots with the specified number of flights

Number of flights	Number of pilots
1	3
2	4
3	6
4	10
5	1
6	6
7	2
8	0
9	2
10	1

It can be seen that the goal of at least 20 pilots with at least 4 flights was reached

### Flight parameters

#### Duration of flight

Data on the duration of flights were available for all 145 flights; flight time ranged from 48 to 160 min, with a median (quartiles) of 86 (76; 99) min.

#### Altitude

Data on flight altitude were also available in 145 flights. As basic characteristic, the maximum altitude per minute was chosen. The median value of these per-minute maxima was computed for each flight. These values ranged from 3,464 to 42,135 ft, varying around a median (quartiles) of 21,540 (15,399; 27,032) ft. In addition, the maximum value of the per-minute maxima per flight was computed, which ranged from 6,070 to 46,134 ft, varying around a median (quartiles) of 30,970 (24,415; 40,485) ft.

#### Distribution of G-forces

In 144 flights, a G_z_-protocol was available comprising minima and maxima reached per minute. The median of the per-minute maxima within each flight showed a range of 1.14 to 4.09 G_z_, with a median (quartiles) of 1.68 (1.44; 2.08) G_z_. As second measure, the maximum of the per-minute maxima per flight was chosen, which ranged from 2.89 to 9.24 G_z_, with a median (quartiles) of 7.33 (5.83; 8.04) G_z_. As the pressure breathing system was activated at 4 G_z_, we also determined the number of minutes per flight during which more than 4 G_z_ were reached. The median (quartiles) frequency per flight was 11 (6; 16), with a minimum of 0 and a maximum of 28.

Based on the median values of these exposure variables, additional binary exposure variables per flight were defined, indicating either a flight duration above 86 min, a maximum altitude above 30,000 ft, a maximum G_z_-value above 7.33, or a number of minutes above 4 G_z_ of more than 11 times per flight.

### Outcome variables

#### Symptoms

Amongst the 9 symptoms comprising difficulty of breathing, earache, headache, eye discomfort, cough, sputum, scratchy throat, dry mouth, dry nose, there were no statistically significant differences when comparing values obtained before and after flights. Symptom scores were always very low, with mean values never exceeding 10% of the full 100 mm VAS scale.

#### FeNO

**Fig. 1:**
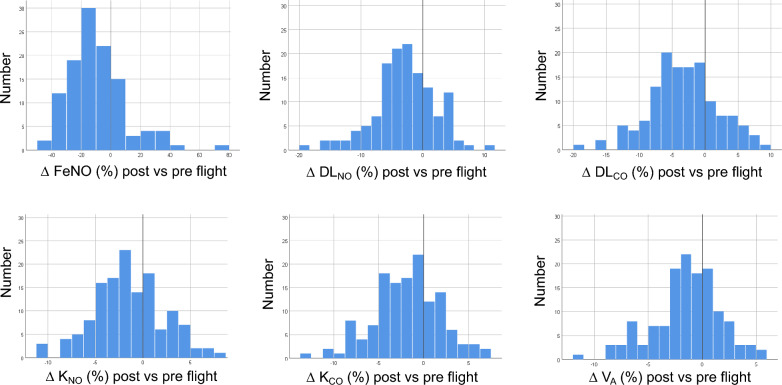
**A**–**F** Distribution of the percent changes post-flight minus pre-flight of FeNO (panel **A**), DL_NO_ (panel **B**), DL_CO_ (panel **C**), K_NO_ (panel **D**), K_CO_ (panel **E**) and V_A_ (panel **F**). *FeNO* fractional concentration of exhaled nitric oxide, *DL*_*NO*_ diffusing capacity for NO, *DL*_*CO*_ diffusing capacity for CO, *K*_*NO*_ diffusing capacity for NO per volume V_A_, *K*_*CO*_ diffusing capacity for CO per volume V_A_, *V*_*A*_ alveolar volume. In each panel, the value of zero is marked by a vertical line. All changes were significantly different from zero (see text)

Overall, 113 pairs of FeNO values were available that satisfied the quality criteria and could be used for analysis. Geometric mean values (geometric SD) and factors of change are given in Table 3. FeNO significantly (*p* < 0.001) decreased after the flights, as illustrated in Fig. 1A via the distribution of percent changes. On average, the magnitude of changes was proportional to the magnitude of baseline values of FeNO, suggesting a proportional response; this could be demonstrated by regression analysis of the logarithm of relative changes versus log baseline values. Moreover, changes did not show significant differences between flights performed before noon, in the afternoon, or in the evening.

**Table 3:** Mean values and standard deviations of outcome variables DL_NO_, DL_CO_, K_NO_, K_CO_ and V_A_ measured pre- and post-flights and of the corresponding percent changes post minus pre

Variable [unit]	Pre	Post	Delta or *ratio	95% CI of delta or *ratio	Minimum of delta or *ratio	Maximum of delta or *ratio	*p* value post vs pre
FeNO* [ppb]	17.9 (1.88)	15.8 (1.90)	0.889 (1.22)	0.857; 0.923	− 11.9	13.0	** < 0.001**
DL_NO_ [mmol × min^−1^ × kPa^−1^]	50.27 ± 5.92	48.72 ± 5.58	– 2.90 ± 5.01	–3.75; -2.05	− 18.9	11.7	** < 0.001**
DL_CO_ [mmol × min^−1^ × kPa^−1^]	11.03 ± 1.35	10.65 ± 1.28	– 3.24 ± 5.18	– 4.12; -2.36	− 18.7	9.0	** < 0.001**
K_NO_ [mmol × min^−1^ × kPa^−1^ × L^−1^]	7.90 ± 0.73	7.77 ± 0.71	– 1.49 ± 3.71	– 2.12; -0.87	− 10.9	7.7	**0.001**
K_CO_ [mmol × min^−1^ × kPa^−1^ × L^−1^]	1.73 ± 0.18	1.70 ± 0.18	– 1.91 ± 3.71	– 2.54; -1.28	− 13.0	6.8	** < 0.001**
V_A_ [L]	6.37 ± 0.60	6.28 ± 0.62	– 1.40 ± 3.11	– 1.92; -0.87	−11.5	5.6	** < 0.001**
DL_NO_/DL_CO_* [ratio]	4.56 (1.05)	4.58 (1.05)	1.004 (1.03)	0.998; 1.009	0.93	1.09	0.336

^*^Regarding FeNO and DL_NO_/DL_CO_ ratio post/pre are given, with geometric mean and standard deviation (in parentheses, to be interpreted as variability factor of the geometric mean). *FeNO*  fractional concentration of exhaled nitric oxide (NO), *DL*_*NO*_  diffusing capacity for NO, *DL*_*CO*_  diffusing capacity for CO, *K*_*NO*_  diffusing capacity for NO per volume V_A_, *K*_*CO*_  diffusing capacity for CO per volume V_A_, *V*_*A*_  alveolar volume. Statistical comparisons were performed with a mixed ANOVA model comprising pilots as random factor and pre vs post as fixed factor, including an interaction between both factors that was never statistically significant. Significant (*p* < 0.05) changes are marked in boldface

#### Diffusing capacity for CO and NO

There were 136 valid pairs of measurements of combined diffusing capacity. Mean and SD of absolute values and their changes are given in Table 3. Compared to the values before the flights, those after the flights were significantly lower (*p* ≤ 0.001 each) for DL_NO_, DL_CO_, K_NO_, K_CO_ and V_A_ but not for the ratio DL_NO_/DL_CO_. The distribution of the corresponding percent changes is illustrated for DL_NO_, DL_CO_, K_NO_, K_CO_ and V_A_ in Fig. 1B–F. There were no significant differences in these changes between flights performed in the morning, afternoon, or evening. There were no significant differences between the percent changes of DL_NO_ and DL_CO_, or those of K_NO_ and K_CO_; however, the percent changes of K_NO_ were smaller than those of DL_NO_, and those of K_CO_ were smaller than those of DL_CO_ (*p* < 0.001 each).

#### Urine samples

Matching pre- and post-flight urine samples were available in 138 cases, with 61 referring to flights before noon, 65 to afternoon flights, and 12 to evening flights. Of these datasets, 99 fulfilled the quality criteria (see Methods). Overall, the mean ± SD concentrations of the ratio of 8-OHdG over creatinine were 167,176 ± 59,379 pg/g pre-flight and 181,964 ± 60,894 pg/g post-flight and significantly different (*p* = 0.003), the mean ± SD percent increase being 15.7 ± 37.8 (95% CI: 8.2, 23.3) %. As urine sampling from previous flights on the same day might influence the values, flights performed in the morning were further analyzed separately (*n* = 46); in these flights, the ratio of 8-OHdG and creatinine (*p* < 0.001) increased by 25.4 ± 34.4% (95% CI: 15.2, 35.7; *p* < 0.001).

### Dependence on time of day and repetition of flights on different days or the same day

On 24 occasions, the same pilot performed two flights on the same day, one before and one after noon. Measurements prior to the second flight were performed 2–3 h after the measurements performed after the first flight. The values of FeNO, DL_NO_, DL_CO_ and V_A_ prior to the second flight were not significantly different from those prior to the first flight. The same was true when comparing the changes in DL_NO_, DL_CO_ and V_A_ occurring during the flights.

We also compared pre-flight values performed on different days by the same pilots, using repeated-measures ANOVA. If one pilot had two flights on 1 day, the first one was chosen for this analysis. There were no statistically significant changes over time in FeNO, DL_NO_, DL_CO_ and V_A_, i.e., despite the slight reductions observed after the flights, baseline values had been restored at the following days.

### Relationship between outcome variables and flight parameter

First, we determined the dependence on the single flight parameters listed above, using mixed-model ANOVA for the post–pre differences. This was done with the flight parameters taken as continuous variables or as binary exposure variables as defined above. The latter, simplified approach was chosen to facilitate interpretation and practical conclusions.

#### FeNO

**Fig. 2:**
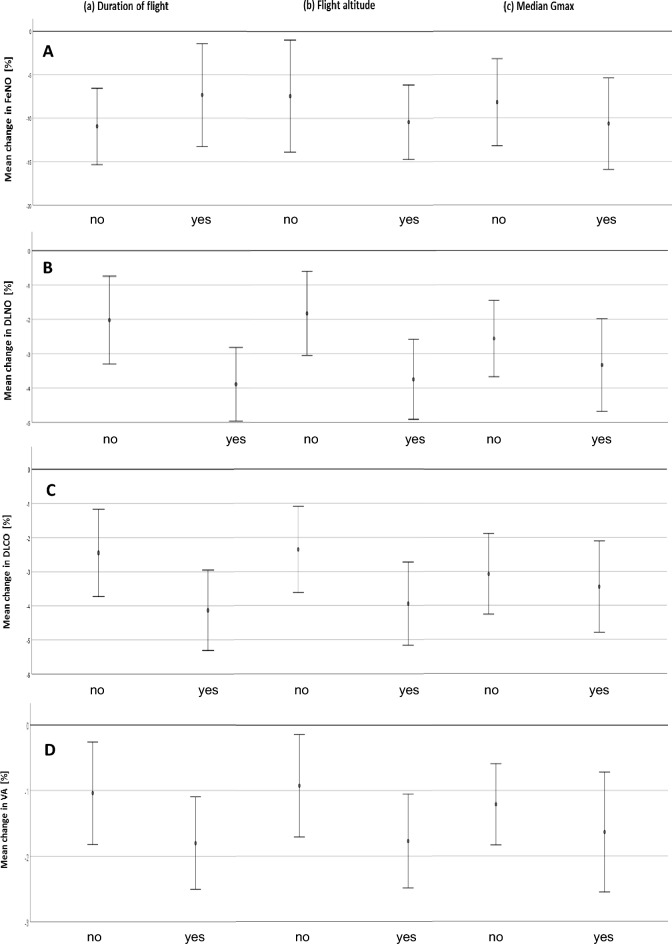
**A**–**D** Dependence of the percent changes of outcome variables on binary exposure parameters according to: (a) flight duration: yes = above 86 min, (b) maximum flight altitude: yes = above 30,000 ft; (c) per-minute maxima of G_z_: yes = 4 G_z_ more often than 11 times per flight. Panel **A**: FeNO = fractional concentration of exhaled nitric oxide, Panel **B**: DL_NO_ = diffusing capacity for NO, Panel **C**: DL_CO_ = diffusing capacity for CO, Panel **D**: V_A_ = alveolar volume. Estimates from mixed model ANOVA and their 95% confidence intervals are shown

The log change in FeNO was not significantly dependent on the duration of the flight, the median or maximum flight altitude, or the median or maximum G_z_-force, or the number of minutes during which 4 G_z_ was exceeded. There was, however, a significant difference in FeNO levels between pilots (*p* < 0.05). When using the binary exposure variables as defined above, similar results were obtained. Figure 2A shows the percent changes in FeNO as a function of the three binary exposure variables comprising duration, maximum altitude, and frequency of minutes above 4 G_z_.

#### Diffusing capacity for CO and NO

When using the single exposure parameters, the percent changes in DL_NO_ were not significantly dependent on the duration of flight, median or maximum flight altitude, number of minutes above 4 G_z_, and median or maximum of maximum G_z_. This was true for both the continuous and the binary exposure variables. However, for the binary variable of maximum flight altitude above 30,000 ft, there was a tendency towards a greater decrease in DL_NO _(*p* = 0.071). Regarding K_NO_, no significant associations with exposure parameters were found.

The percent changes in DL_CO_ and K_CO_ were not significantly dependent on the exposure parameters in the form of continuous or binary variables. For the binary variable of maximum flight altitude above 30,000 ft, there was again a tendency towards a greater decrease of DL_CO_ (*p* = 0.092). K_CO_ showed a tendency of depending on the maximum of maximum per-minute G_z_-level (*p* = 0.086) as continuous variable and on the median of maximum G_z_-values (*p* = 0.090).

The percent changes of V_A_ did not show statistically significant dependences on the continuous or binary exposure variables, although there was a tendency towards more reduction with increasing flight duration (*p* = 0.063) as continuous variable. In most of these analyses, the percent changes in V_A_ showed significant differences between pilots (*p* < 0.05). The changes in DL_NO_, DL_CO_ and V_A_ are illustrated in Fig. 2B–D as functions of the binary exposure variables regarding flight duration, maximum altitude and frequency of minutes above 4 G_z_.

#### 8-OHdG

According to mixed ANOVA models, the pre–post flight changes of the ratio of 8-OHdG over creatinine were not significantly dependent on any of the four single flight exposure variables.

### Combination of exposures

Second, we combined the binary exposure variables using either two or three of these variables and analyzed the dependence of outcome variables on these combinations. The combination was defined as follows: if at least one variable was in the high category, the combined score also was in the high category. Thus, the combined score assumed the low category only if all respective flight parameters were in the low category. In additional analyses, we also had defined additive sum scores ranging from 0 to 2 or 3, but there were never significant differences between the different categories above the lowest category; thus, we used the simplified combined scores comprising only two categories. As for the single exposure variables, the post–pre percent differences of outcome measures were analyzed using mixed ANOVA models.

**Fig. 3:**
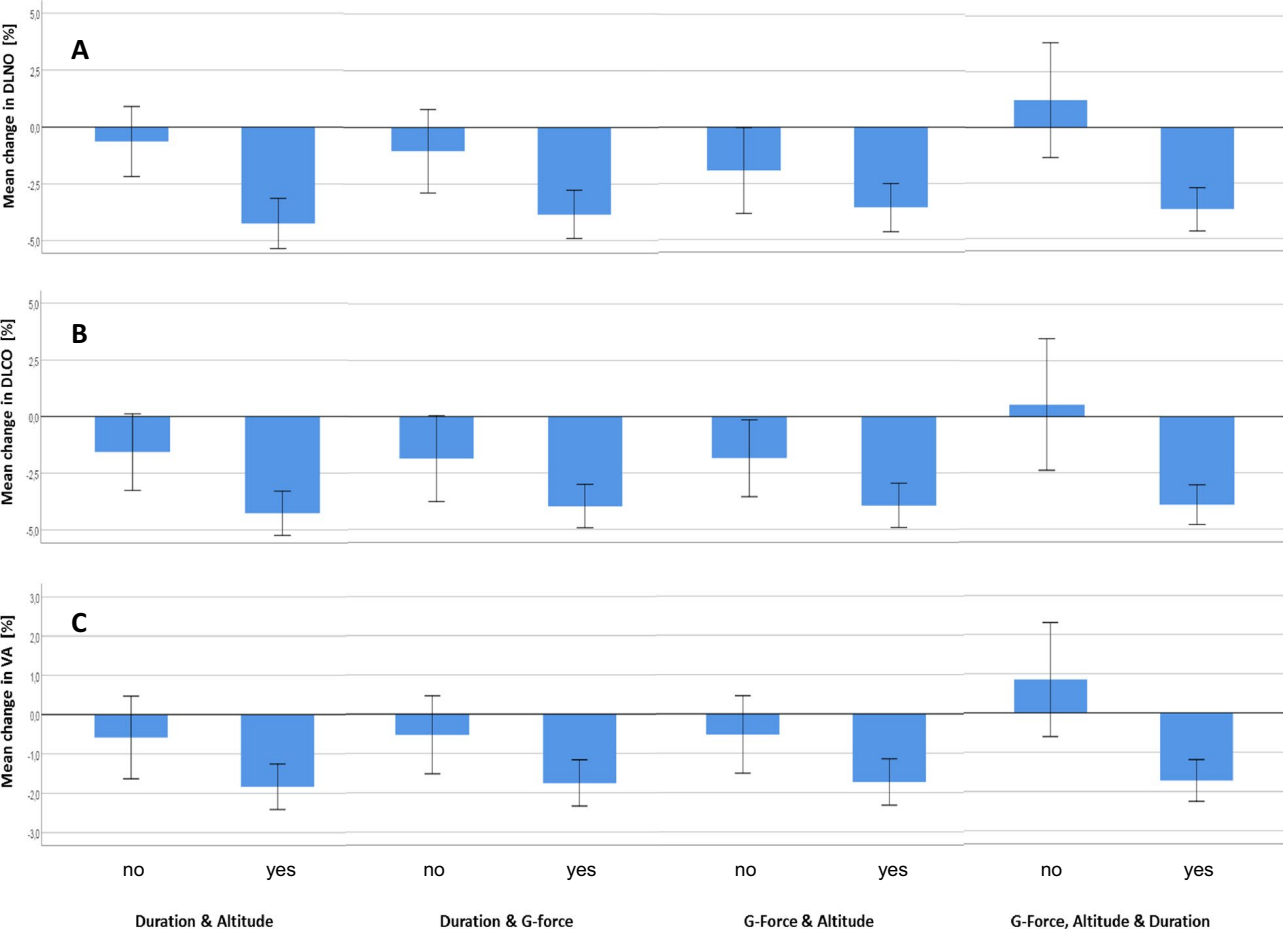
**A**–**C** Effects in terms of percent changes of outcome variables of diffusing capacity as a function of combinations of the binary exposure parameters used in Fig. 2. Duration and Altitude: flight duration above 86 min and/or maximum flight altitude above 30,000 ft. Duration and G-force: flight duration above 86 min and/or 4 G_z_ more than 11 times per flight. G-force and Altitude: 4 G_z_ more than 11 times per flight and/or maximum flight altitude above 30,000 ft. G-force, Altitude and Duration: 4 G_z_ more than 11 times per flight and/or maximum flight altitude above 30,000 ft and/or flight duration above 86 min. Panel **A**: DL_NO_ = diffusing capacity for NO, Panel **B**: DL_CO_ = diffusing capacity for CO, Panel **C**: V_A_ = alveolar volume. Estimates from mixed model ANOVA and their 95% confidence intervals are shown

The combined exposure variables did not show significant associations with FeNO, just as the single variables. The results for diffusing capacity are given in Table 4. They indicate that only the combination of flight duration and altitude reached statistical significance (*p* < 0.05) for DL_NO_ and DL_CO_. There were no associations when G_z_-force was combined with either flight duration or altitude. The triple combination also was informative regarding DL_CO_ and V_A_; however, it appeared that the inclusion of the G_z_-forces into the combination deteriorated the degree of associations. To illustrate the results, the percent changes associated with the combined binary flight parameters are shown in Fig. 3A–C for DL_NO_, DL_CO_ and V_A_.

**Table 4:** Dependence of indices of diffusing capacity indices from combinations of binary exposure variables obtained by mixed model ANOVA (see text)

Combination of exposure parameters	Outcome parameters	FeNO	DL_NO_	K_NO_	DL_CO_	K_CO_	V_A_
Duration and altitude low: *n* = 40 high: *n* = 96	Estimate [%]	3.26 ± 4.65	− 2.48 ± 1.02	− 1.44 ± 0.74	− 2.38 ± 1.04	− 1.33 ± 0.75	− 1.00 ± 0.60
	95% CI [%]	− 6.00; 12.51	− 4.49; − 0.47	− 2.91; 0.03	− 4.45; − 0.31	− 2.81; 0.15	− 2.20; 0.19
	*p* value	0.486	**0.016**	0.054	**0.025**	0.077	0.099
Duration and G-Force low: *n* = 28 high: *n* = 108	Estimate [%]	2.94 ± 4.88	− 1.65 ± 1.16	− 0.82 ± 0.84	− 1.50 ± 1.19	− 0.69 ± 0.85	− 0.77 ± 0.68
	95% CI [%]	− 6.77; 12.65	− 3.95; 0.65	− 2.49; 0.85	− 3.86; 0.86	− 2.37; 0.99	− 2.12; 0.58
	*p* value	0.548	0.159	0.332	0.211	0.417	0.259
G-Force and Altitude low: *n* = 28 high: *n* = 108	Estimate [%]	− 5.18 ± 4.71	− 1.46 ± 1.15	− 0.58 ± 0.83	− 1.37 ± 1.18	− 0.33 ± 0.84	− 1.09 ± 0.67
	95% CI [%]	− 14.55; 4.19	− 3.74; 0.81	− 2.22; 1.07	− 3.70; 0.96	− 1.99; 1.32	− 2.41; 0.24
	*p* value	0.274	0.205	0.489	0.246	0.691	0.106
G-Force and Altitude and Duration low: *n* = 13 high: *n* = 123	Estimate [%]	3.67 ± 6.75	− 2.90 ± 1.55	− 0.91 ± 1.14	− 3.20 ± 1.59	− 1.19 ± 1.14	− 1.98 ± 0.90
	95% CI [%]	− 9.76; 17.10	− 5.98; 0.18	− 3.16; 1.35	− 6.35; − 0.06	− 3.45; 1.07	− 3.77; − 0.19
	*p* value	0.588	0.065	0.426	**0.046**	0.299	**0.030**

Exposure variables were defined from the three basic exposure parameters: flight duration (≤ 86 min vs > 86 min), maximum altitude (≤ 30,000 ft vs > 30,000 ft), and ≤ 11 vs > 11 occurrences of at least 4 G_z_-events during the flight. The combined exposures were also binary and defined via the requirement that at least one of the two or three, respectively, exposure parameters was in the higher category, i.e., the reference of each combined exposure reflected low exposure in all single parameters of the combination. Results are given as mean and SEM of the difference of percent changes post- versus pre-flight, whereby the low value of the combined exposure parameter served as reference. *DL*_*NO*_  diffusing capacity for NO, *DL*_*CO*_  diffusing capacity for CO, *K*_*NO*_  diffusing capacity for NO per volume V_A_, *K*_*CO*_  diffusing capacity for CO per volume V_A_, *V*_*A*_  alveolar volume. Significant (*p* < 0.05) effects are marked in boldface

#### 8-OHdG

Similar to the single binary exposure variables, the change in the ratio of 8-OHdG over creatinine was not significantly dependent on the binary twofold combinations and the threefold combination of these variables.

## Discussion

This study analyzed changes in lung function occurring after usual training flights in a typical tactical fighter aircraft under real-life conditions. Outcome measures were the concentration of exhaled nitric oxide (FeNO), the lung diffusing capacity for nitric oxide (DL_NO_) and carbon monoxide (DL_CO_), alveolar volume (V_A_), and the concentration of 8-OHdG in urine. In 145 flights performed by 35 pilots, statistically significant reductions in FeNO, DL_NO_, DL_CO_ and V_A_, and a significant increase in the level of 8-OHdG occurred. When comparing repeated flights, baseline values prior to the first flight were restored before the second flight. Amongst the parameters that either directly described G_z_-forces, or indirectly the action of the positive pressure breathing and oxygenation support, the combination of long flight duration and high altitude appeared to be linked to greater changes in DL_NO_ and DL_CO_, whilst there were no associations with G_z_-forces. There were also no significant associations between flight parameters and FeNO or 8-OHdG. Importantly, we found no indication of persistent effects in flights repeated on subsequent days. Taken together, all observed changes were small and would not be considered as relevant from a clinical perspective [15, 25–27]. However, they demonstrated the power of the non-invasive procedures for the detection of very small physiological effects under real-life conditions. In view of this, there seemed to be no need for specific countermeasures to prevent these effects, independent of the efforts towards steady improvement of pressure-suits and other equipment.

We used the fractional concentration of exhaled nitric oxide (FeNO) as a potential indicator of stress exerted on the bronchial mucosa. The exhaled NO primarily originates from the bronchi, and the contribution from the alveoli is very small [28]. FeNO depends not only on bronchial NO production but also on properties of the mucosa including its ability to transport NO and the presence of oxidants as potential scavengers, thereby rendering FeNO informative beyond its common use in asthma diagnostics [29]. This is particularly true when considering that changes linked to Th2-like immune responses need more time than the short interval between the flight and the assessments in this study [30]. If the transport barrier should be disturbed by, for example, increased mucus production as a response to inhalation of dry air, FeNO would probably decrease, similar to one of the mechanisms underlying reduced FeNO in cystic fibrosis [31]. The same would happen with increased production of oxygen radicals, possibly due to oxygen from the supply system of the aircraft [32]. Assuming that the endogenous NO production did not change within approx. 2 h, the observed reductions in FeNO pointed towards these two mechanisms. We thus expected a correlation with the duration of the flight but possibly the minimum duration of 48 min was already long enough to elicit the effects so that there was no additional change with increasing flight duration. Symptom scores, especially those of the upper airways, were low before and after the flights and there was no correlation with FeNO. The pilots with a history of respiratory allergy showed, on average, the same relative reductions of FeNO as those without such a history, i.e., not only their baseline FeNO values were higher but also the magnitude of change. This is consistent with the interpretation that the observed changes in FeNO were due to alterations in the airway mucosa that were related to the flight conditions applying to all pilots, such as the inhalation of dry air, but not influenced by allergic responses elicited by the flights. It also should be pointed out that the pilots with a history of respiratory allergy did not report corresponding respiratory symptoms of any kind prior to the flights or afterwards and that all of them had undergone allergen-specific immunotherapy (desensitization) in their career.

Diffusing capacity of the lung was assessed for three purposes. First, to determine the alveolar volume V_a_ via helium dilution. The aim was to detect potential reductions of the volume accessible to gas transport, possibly due to atelectasis, either attributable to the action of G-forces or the resorption of high-oxygen breathing gas [33]. Indeed, V_A_ decreased after the flights, suggesting that at least one of these mechanisms was involved.

Second, DL_NO_ and K_NO_ were chosen as outcome variables, since they are capable of indicating changes in the alveolar transport barrier independent from capillary blood volume, and in the case of K_NO_, independent from changes in alveolar volume as long as these are not too large [34, 35]. This is based on the extraordinarily high affinity of NO for haemoglobin, so that the haemoglobin content of the lung becomes secondary [34]. DL_NO_ and K_NO_ significantly decreased after flights, whereby the percent change of K_NO_ was smaller than that of DL_NO_ but still greater than zero. This corresponded to the observation that the percent reduction in V_A_ was smaller than that of DL_NO_ and thus a residual effect regarding K_NO_ and consequently the diffusion barrier remained.

Third, the conventional DL_CO_ and K_CO_ were measured and compared with NO diffusing capacity. DL_CO_ and K_CO_ should be sensitive to additional changes in the available haemoglobin and thus pulmonary capillary volume. The percent changes of DL_CO_ and K_CO_ were slightly larger than those of DL_NO_ and K_NO_, respectively, suggesting a tiny additional effect on the vascular bed, through either G-forces or oxygenation. However, about 3 h prior to the post-measurement, the pre-assessment of DL_CO_ involving inhalation of CO had been performed, and this inhalation might have been responsible for the difference. In the tight schedule, we did not determine the levels of carboxyhemoglobin or exhaled CO for the calculation of correction factors of DL_CO_. In previous exposure studies [24, 36], however, we had found that for time intervals of slightly more than 2 h the correction of DL_CO_ and K_CO_ was about 0.7%. A similar effect might explain that in the present data the reductions of the uncorrected CO diffusing capacity were slightly larger than those of NO diffusing capacity. Thus, conclusions on additional effects regarding the vascular bed are hypothetical.

Oxidative stress was assessed via the determination of oxidized guanosine species in urine. To be sensitive to a spectrum of changes, we used a kit comprising the several related molecular species, amongst them the well-known 8-hydroxy-2’-deoxyguanosine (8-OHdG) [14, 37], which we took *pars pro toto* for this type of alteration. Whilst the concentrations of both, 8-OHdG and creatinine, decreased after the flights, the decrease in creatinine levels was stronger, leading to a significant increase in the ratio of 8-OHdG and creatinine. This increase was larger when restricting our analysis to flights before noon, i.e., without previous flights on that day and/or potentially variable pre-flight behaviour of the pilots. Whether the slight increase in normalized 8-OHdG was due to the general stress exerted by the flight or should be specifically attributed to the inhalation of oxygen, cannot be derived from our findings. The return to baseline values within the same day suggested that the observed change in normalized 8-OHdG had no further clinical implications.

There were no significant associations with flight parameters, either as single indices or in combination.

Differences between pilots were apparent regarding absolute FeNO values, as some of them had a history of allergic rhinitis, although without symptoms during the time of the study. There were also differences in absolute values of alveolar volume and other parameters of diffusing capacity that are naturally related to height and body size [25]. In the analyses addressing the associations between flight parameters and percent changes, there also appeared differences between pilots, some of them showing virtually no changes and others markedly larger than average changes, as indicated by the minima and maxima in Table 3. Despite similar ranges, the association with the pilots was, however, statistically significant only for alveolar volume, possibly because this volume was determined via helium dilution only, i.e., without involvement of NO and CO, and could be measured with the highest accuracy.

Previous data from studies performed in centrifuges [38] have already demonstrated effects of high G-forces on the lung, especially on lung volumes. Centrifuges have the advantage of providing highly standardized and reproducible exposure conditions, but most available studies involved variations in only one type of exposure parameters, not in three of them (duration, altitude, G_z_-force) and with support systems. The novelty of our findings is the assessment under real-flight conditions comprising the whole variety of flight characteristics encountered in training scenarios. This was possible owing the ability to incorporate measurements into routine flight schedules. It is also relevant that the fighter jet used by the squadron, the Eurofighter Typhoon, is a typical high-performance fighter aircraft of the fourth generation, with capabilities similar to those of other aircrafts that are in international use for similar purposes [39, 40]. Assuming that the differences in flight characteristics and support systems between jets are secondary compared to the requirement to master the challenge from exposures, the present results should be applicable to other airplanes, too. Thus, the results are of interest not only from a physiological point of view, as the flights provided a unique opportunity for real-life physiological challenges, but also from the perspective of occupational medicine.

### Limitations of the study

The study had the limitation that we could not implement further assessments including sampling from the respiratory tract and blood draws, due to their invasive nature and the restrictions of the time schedule as well as safety reasons. This prevented us from assessing inflammatory responses, local lipid damage or antioxidant enzyme profiles. We also had to estimate the correction factor regarding carboxyhemoglobin and the assessment of DL_CO_ after the flights from previous studies. Although 22 pilots performed at least 4 flights and one pilot even 10 flights, a higher number of pilots with a high number of flights might have allowed to identify systematic differences between pilots regarding their response pattern. The delay between the end of the flight and the measurements was about 28 min on average, with a minimum of 13 min, and this delay might already have been long enough for some acute effects to disappear, especially regarding micro-atelectasis [33] or oxygen-induced effects on pulmonary capillaries. Despite this, we still found suggestions of atelectasis from the changes in V_A_. From a clinical perspective, it might be argued that effects disappearing in very short time bear a lower potential to become clinically relevant over long time than more persistent effects. However, a definitive exclusion of long-term effects would require a follow-up study over several years.

## Conclusions

Using measurements before and after typical training flights in a typical fourth-generation fighter aircraft under real-flight conditions, we observed changes in a number of parameters that could be determined non-invasively without interference with the flights. Post-flight assessments were performed on average 28 min after landing, and several changes were observed. The reductions in the diffusing capacities for NO and CO argue for both, an effect arising from atelectasis and an increase of diffusion barrier, but without changes in capillary blood volume. The decrease in exhaled endogenous NO implies bronchial mucosal irritation or local oxidative stress, and the increase in urinary oxidized guanosine species normalized by creatinine suggests systemic oxidative stress. Although all changes were small and not clinically relevant [41], they demonstrated the occurrence of physiological effects from real training flights in a modern fighter jet that were detectable by sensitive, non-invasive procedures.

### Supplementary Information

**Additional file 1: Figure S1.** First, at least two measurements of FeNO were performed, until the quality criteria (plateau values need to agree within 10% of each other) were fulfilled. Second, two measurements of diffusing capacity took place. A time interval of 4 min between the two measurements was chosen to ensure washout of previously inhaled gas mixture. This period was used to answer the symptom questionnaire. After completion of the functional tests the pilots provided a urine sample for the measurement of 8-OHdG.

## Data Availability

The datasets generated and analyzed during the current study are not publicly available due to military safety reasons, but available upon reasonable request.

## Abbreviations

g: Unit of gravitational acceleration
G_z_: Acceleration forces directed towards the head
DL_CO_: Diffusing capacity for carbon monoxide
DL_NO_: Diffusing capacity for nitric oxide
NO: Nitric oxide
CO: Carbon monoxide
V_A_: Alveolar volume
K_CO_: Diffusing capacity for carbon monoxide/alveolar volume
K_NO_: Diffusing capacity for nitric oxide/alveolar volume
VC: Vital capacity
FeNO: Fractional concentration of exhaled nitric oxide
8-OHdG: 8-Hydroxy-2’-deoxyguanosine
SD: Standard deviation
PPB: Positive pressure breathing
PBG: Pressure breathing for G-protection
ECS: Eurofighter environmental control system
G-LOC: G-induced loss of consciousness
ATS/ERS: American Thoracic Society/European Respiratory Society
SEM: Standard errors of mean
VAS: Visual analogue scale
