# Supplementary material for: Effects of training flights of combat jet pilots on parameters of airway function, diffusing capacity and systemic oxidative stress, and their association with flight parameters
Source: Eur J Med Res. 2024 Feb 5;29:100. doi: 10.1186/s40001-024-01668-z (PMC10840181; doi:10.1186/s40001-024-01668-z)
Supplement: Supplementary file 1 — Additional file 1: Figure S1. First, at least two measurements of FeNO were performed, until the quality criteria (plateau values need to agree within 10% of each other) were fulfilled. Second, two measurements of diffusing capacity took place. A time interval of 4 min between the two measurements was chosen to ensure washout of previously inhaled gas mixture. This period was used to answer the symptom questionnaire. After completion of the functional tests the pilots provided a urine sample for the measurement of 8-OHdG. [file 40001_2024_1668_MOESM1_ESM.pptx]

## Slide 1
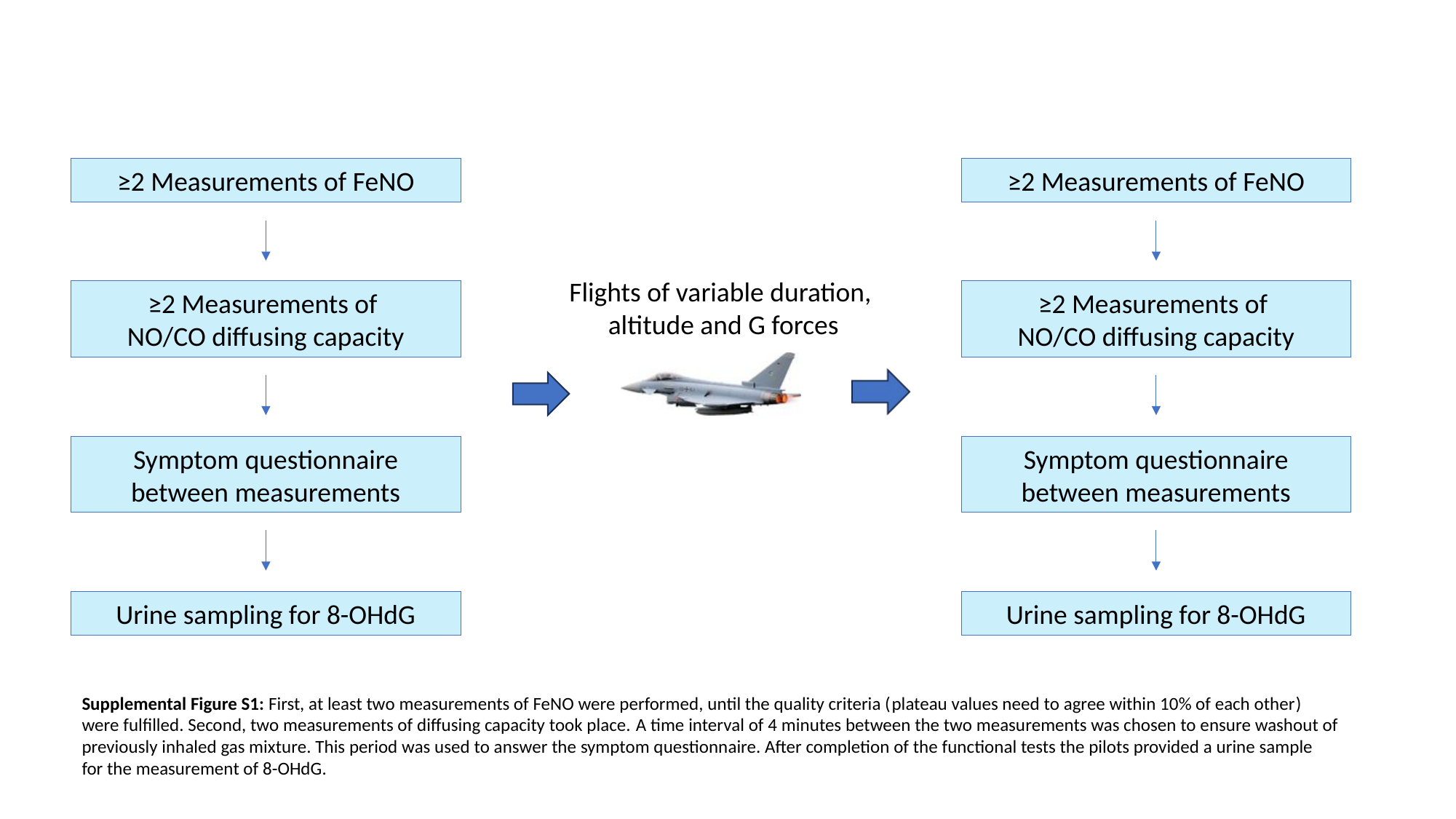

≥2 Measurements of FeNO
≥2 Measurements of FeNO
Flights of variable duration,
 altitude and G forces
≥2 Measurements of
NO/CO diffusing capacity
≥2 Measurements of
NO/CO diffusing capacity
Symptom questionnaire
between measurements
Symptom questionnaire
between measurements
Urine sampling for 8-OHdG
Urine sampling for 8-OHdG
Supplemental Figure S1: First, at least two measurements of FeNO were performed, until the quality criteria (plateau values need to agree within 10% of each other) were fulfilled. Second, two measurements of diffusing capacity took place. A time interval of 4 minutes between the two measurements was chosen to ensure washout of previously inhaled gas mixture. This period was used to answer the symptom questionnaire. After completion of the functional tests the pilots provided a urine sample for the measurement of 8-OHdG.
